# Cardiac-specific renalase overexpression alleviates CKD-induced pathological cardiac remodeling in mice

**DOI:** 10.3389/fcvm.2022.1061146

**Published:** 2022-12-15

**Authors:** Yi Wang, Linnan Bai, Jiejun Wen, Fangfei Zhang, Sijie Gu, Feng Wang, Jianyong Yin, Niansong Wang

**Affiliations:** ^1^Department of Nephrology, Shanghai Sixth People’s Hospital, Shanghai Jiao Tong University School of Medicine, Shanghai, China; ^2^Department of Nephrology, Sir Run Run Shaw Hospital, Zhejiang University School of Medicine, Hangzhou, China

**Keywords:** renalase, pathological cardiac remodeling, CKD, cardiac fibrosis, cardiac fibroblasts

## Abstract

**Introduction:**

CKD-induced pathological cardiac remodeling is characterized by myocardial hypertrophy and cardiac fibrosis. The available therapeutic options are limited, it is thus urgently needed to identify novel therapeutic targets. Renalase (RNLS) is a newly discovered protein secreted by the kidney and was found beneficial in many renal diseases. But whether it exerts protective effects on cardiac remodeling in CKD remains unclear.

**Methods:**

RNLS knockout (KO) and wild-type (WT) mice were both used to build CKD models and the adeno-associated virus (AAV9) system was used to overexpress RNLS cardiac specifically. Echocardiography was performed to detect cardiac structural changes every 6 weeks until 18 weeks post-surgery. High throughput sequencing was performed to understand the underlying mechanisms and the effects of RNLS on cardiac fibroblasts were validated *in vitro*.

**Results:**

Knockout of RNLS aggravated cardiac remodeling in CKD, while RNLS cardiac-specific overexpression significantly reduced left ventricular hypertrophy and cardiac fibrosis induced by CKD. The following RNA-sequencing analysis revealed that RNLS significantly downregulated the extracellular matrix (ECM) receptor interaction pathway, ECM organization, and several ECM-related proteins. GSEA results showed RNLS significantly downregulated several profibrotic biological processes of cardiac fibroblasts which were upregulated by CKD, including fibroblast proliferation, leukocyte migration, antigen presentation, cytokine production, and epithelial-mesenchymal transition (EMT). *In vitro*, we validated that RNLS reduced the primary cardiac fibroblast proliferation and α-SMA expression stimulated by TGF-β.

**Conclusion:**

In this study, we examined the cardioprotective role of RNLS in CKD-induced cardiac remodeling. RNLS may be a potential therapeutic factor that exerts an anti-fibrotic effect in pathological cardiac remodeling.

## Introduction

Cardiovascular disease (CVD) is a major cause of morbidity and mortality in patients with chronic kidney disease (CKD) (1), accounting for 58% of deaths in individuals with end-stage renal disease (ESRD) after adjusting for age and sex (2). Two primary characteristics of pathological cardiac remodeling in CVD caused by CKD are left ventricular hypertrophy (LVH) (3) and extensive fibrosis (4), which both are strong predictors of cardiac sudden death in maintenance hemodialysis patients (5–7). Unfortunately, current treatment strategies, including hemodialysis and peritoneal dialysis, cannot adequately prevent or correct these abnormalities. Therefore, defining new treatments is urgently required. Several studies have found that patients who underwent kidney transplantation attained improvements in cardiac function, including regression in LVH (8–10). This evidence suggests that kidney-associated factors may be the breakout direction of finding novel therapeutic targets for cardiac complications in CKD.

Renalase (RNLS) is one such factor, which was identified in 2005 by the research team of Gary V. Desir (11). It is highly expressed in the kidney and heart, as well as in the liver, skeletal muscles, pancreas, and small intestines (11–15). In the kidney, RNLS can be secreted into blood and urine by renal proximal tubular epithelial cells (16). It is generally accepted that renal RNLS expression markedly decreased in animal models of CKD (17, 18). Human renal biopsy RNA-seq data from the GEO database also showed that RNLS expression significantly decreased in tubule interstitium (where RNLS is secreted) from patients with primary renal diseases of CKD (Supplementary Figure 1). Desir et al. found that RNLS was barely detectable in plasma from patients with ESRD (11), and the renal and plasma levels were correlated with renal functions (19, 20). In their recent research, total human plasma RNLS levels were correlated positively with the estimated glomerular filtration rate (GFR) and negatively with the estimated GFR stage (I–IV) in 267 CKD patients (20). These findings implied that the reduced RNLS expression may involve in CKD complications, but more evidence is needed to confirm this hypothesis.

Many studies showed that RNLS could modulate the severity of acute injuries in renal or cardiac diseases (21–26). Some past studies considered RNLS as a beneficial factor for the cardiovascular system, mainly ascribed to its possible ability to metabolize catecholamines (11, 18, 19), but this view has been questioned by the present findings (27–29). To examine whether RNLS has beneficial effects in cardio-renal diseases, we conducted a preliminary study in a short-term rat model with subtotal nephrectomy (Nx), and the results showed that the renal function was significantly improved due to the renal overexpression of RNLS via adenovirus (30). Unfortunately, in the previous study, we could not determine whether RNLS exerts protective effects directly on the heart. This question is of great importance for RNLS being a promising therapeutic target in ESRD.

Therefore, to clarify the cardioprotective role of RNLS, we conducted this study by following four key points. (1) We constructed CKD models using both RNLS knockout (KO) and wild-type (WT) mice to confirm what effects RNLS deficiency made on cardiac dysfunction in CKD. (2) To exclude the interferences from the kidney, we concluded RNLS overexpression cardiac-specifically and assessed renal function at mid-treatment and end of treatment. (3) We detected the cardiac structural and functional changes by a long-term observation (up to 18 weeks post-surgery), to demonstrate the protective role of RNLS in chronic cardiac injuries. (4) RNA-sequencing (RNA-seq) analysis was performed to explore the underlying molecular mechanism and the results were validated *in vitro*. Our study sought to confirm the cardioprotective role of RNLS in CKD and provide insights into novel treatments.

## Materials and methods

### Animals and CKD model

Renalase (RNLS) KO mice were generated by Transcription activator-like effector nuclease (TALEN)-mediated gene targeting in C57BL/6J strain mice (Cyagen Biosciences Co. Ltd., Suzhou, China) as described previously (31). Exon 4 of RNLS was selected as the TALEN target site (Supplementary Figure 2). WT (C57BL/6J) mice were sourced from the Nanjing BioMedical Research Institute of Nanjing University. All mice were male and aged 6–8 weeks. The *in vivo* experiments performed in this study were approved by the Institutional Animal Care and Use Committee of Shanghai Jiao Tong University Affiliated Sixth People’s Hospital and were carried out following the appropriate guidelines.

The CKD model was created by a two-step 5/6 nephrectomy (Nx) procedure (32). Briefly, both poles of the left kidney were removed, and the entire right kidney was removed 7 days later (we defined this time point as week 0). 2 weeks after the second procedure, the treatment started. The vector (1 × 10^12^ VG) or AAV9-cTnT-Rnls (1 × 10^12^ VG) was delivered by tail vein injection at a total volume of 100 μL. The AAV9 virus used in this study was synthesized by Hanheng (Shanghai, China). At 18 weeks, the mice were sacrificed. Sham animals underwent the same procedure without any kidney excision. WTSHAM group, *n* = 9; KOSHAM group *n* = 11; WTCKD + vector group, *n* = 10; WTCKD + AAV9-cTnT-Rnls group, *n* = 7; KOCKD + vector group, *n* = 9; KOCKD + AAV9-cTnT-Rnls group, *n* = 8.

### Echocardiography

Transthoracic echocardiography and subsequent quantitative measures were performed at the instrumental analysis center of Shanghai Jiao Tong University with a Vevo 3100 system (FUJIFILM VisualSonics, Toronto, ON, Canada).

### Renal function assessments

At 10 weeks (mid-way through the AAV9 treatment period), renal function was assessed by measuring the transcutaneous glomerular filtration rate (tGFR). The NIC-Kidney device (Mannheim Pharma & Diagnostics GmbH, Mannheim, Germany) was attached to the shaved back of each animal. After a few minutes for collection of baseline values, fluorescein isothiocyanate–labeled sinistrin (7.5 mg/100 g) was injected via the tail vein, and tGFR was calculated based on the kinetics of fluorescence decay (33, 34).

At 18 weeks, blood samples were collected and centrifuged at 3,000 rpm for 15 min to obtain the serum. Serum creatinine was measured by an SYSMES XT-2100i automatic blood cell analyzer and reagents (SYSMES Co., Kobe, Japan).

### Histopathological analysis

Heart tissues were post-fixed overnight in 4% paraformaldehyde, then embedded in paraffin on the embedding station. Heart sections (5 μm) were stained with trichrome for the assessment of cardiac fibrosis and analyzed using ImagePro Plus version 6.0. For immunofluorescence, heart sections were stained with anti–α-myosin heavy chain (MHC) (ABclonal, Wuhan, China), anti–β-MHC (ABclonal, Wuhan, China), anti–wheat germ agglutinin (WGA) (Abcam, Cambridge, UK), anti-collagen Iα (Abcam, Cambridge, UK) and anti–α-smooth muscle actin (SMA) (Abcam, Cambridge).

### RNA-sequencing

Total RNA was extracted using the mirVana microRNA isolation kit (Ambion, Austin, TX, USA), and RNA integrity was evaluated by the Agilent 2100 bioanalyzer (Agilent Technologies, Santa Clara, CA, USA). The libraries were constructed using the TruSeq stranded messenger RNA LTSample prep kit (Illumina, San Diego, CA, USA); then, these libraries were sequenced on the HiSeq 2500 or HiSeq X Ten sequencing platform (Illumina, San Diego, CA, USA) and 125-bp/150-bp paired-end reads were generated. Library preparation and sequencing were performed by OE Biotech Co., Ltd. (Shanghai, China). Results of differential expression analysis are found in the Supplementary material.

### Gene set enrichment analyses

Gene set enrichment analysis (GSEA) was performed by GSEA software 4.1.0 (35, 36). Significant enrichment results were demonstrated based on normalized enrichment score (NES), nominal *p-value* (NOM *p*-val), and the false-discovery rate (FDR). Significant enrichments were required to meet the following three conditions: |NES| > 1, NOM *p*-val < 0.05, and FDR < 0.25.

### Cell culture and treatment

Cardiac fibroblasts (CFs) were isolated from the hearts of neonatal 1- to 3-day-old Sprague–Dawley rats. Briefly, isolated neonatal hearts were minced into small pieces < 1 mm^3^ in size, digested with pancreatin 0.125%, and filtered through 40-μm cell strainers to remove tissue fragments. Cells were plated on plates for 2 h, and attached cells were identified as CFs. CFs were grown in high glucose (4.5 g/l) DMEM containing 10% FBS and 1% antibiotics (penicillin and streptomycin). For protein and RNA isolation, 2 × 10^6^ cells were seeded in 6 cm-culture dishes.

For adenovirus-mediated RNLS transfer, adenovirus overexpressing renalase (Ad-Rnls) or control virus (vector) were transfected into CFs at an MOI of 10 × PFU/cell for 48 h. Adenovirus was synthesized by Hanheng (Shanghai, China). After the overexpression efficiency was evaluated, cells were incubated overnight in a serum-free medium, then cultured in the presence of TGF-β1 (1 ng/ml) for another 24 h, or test cell proliferation by cell counting kit-8 (CCK8) assay.

### Cell counting Kit-8 assay

Cell proliferation was analyzed by CCK8 assay (Dojindo, Kumamoto, Japan) according to the manufacturer’s instructions. Briefly, 2 × 10^3^/100 μL CFs were seeded into 96-well plates and incubated for 0, 24, and 48, 72 h. Then the optical density (OD) value was detected at a wavelength of 450 nm.

### Quantitative real-time polymerase chain reaction (PCR) and western blot

Total RNA was extracted using the FastPure total RNA isolation kit (Vazyme Biotech Co., Nanjing, China) and reverse-transcribed to complementary DNA using HiScript III All-in-one RT SuperMix (Vazyme Biotech Co, Nanjing, China), according to the manufacturer’s instructions. Real-time PCR was performed with SYBER Green PCR Master Mix (Vazyme Biotech Co, Nanjing, China) using a StepOnePlus PCR system (Applied Biosystems, Foster City, CA, USA). Glyceraldehyde-3-phosphate dehydrogenase (GAPDH) was used as an internal normalizer. Primer sequences are noted in the Supplementary materials. Western blot was used to measure α-SMA expression levels in CFs. The primary antibodies were anti-α-SMA (Abcam, Cambridge, UK) and anti–β-tubulin (ABclonal, Wuhan, China).

### Statistical analysis

Data are presented as mean ± standard error of the mean values. All statistical analyses were performed with GraphPad Prism version 8.0 (GraphPad Software, San Diego, CA, USA). A two-tailed unpaired *t*-test was used to determine the differences between the two groups. Statistical significance is indicated by *p < 0.05.

## Results

### RNLS deficiency aggravated pathologic LVH induced by CKD

**FIGURE 1:**
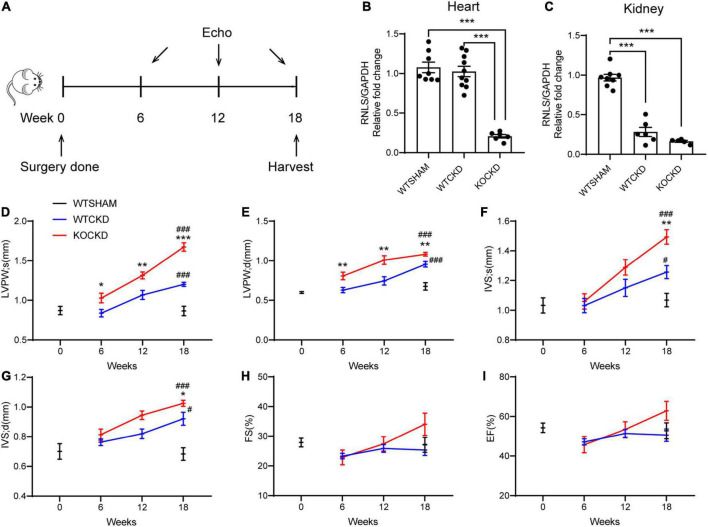
RNLS deficiency aggravated pathologic LVH induced by CKD. **(A)** Design of the experiment. Echocardiography was performed at 6, 12, and 18 weeks after surgery. Mice were sacrificed at 18 weeks. **(B,C)** qPCR analysis of the relative mRNA expression of RNLS in the hearts and kidneys. *n* = 5–10 per group, ***p < 0.001. **(D–I)** Echocardiographic parameters, including LVPWs, LVPWd, IVSs, IVSd, FS, and EF at 0, 6, 12, and 18 weeks. WTSHAM group, *n* = 9; WTCKD group, *n* = 10; KOCKD group, *n* = 9. Data were shown as mean ± SEM. *p < 0.05, **p < 0.01, ***p < 0.001. ^#^ *p* < 0.05, ^###^*p* < 0.001. * vs. WTCKD group, ^#^ vs. WTSHAM group. LVPW, left ventricle posterior wall; IVS, interventricular septum; d, diastole; s, systole; FS, fractional shortening; EF, ejection fraction.

To detect the cardiac structural changes caused by CKD and the effects of RNLS knockout, echocardiography was performed every 6 weeks after surgery (Figure 1A). Hearts and kidneys were harvested at 18 weeks post-surgery. The mRNA expression of RNLS did not change significantly in the hearts of CKD models but markedly decreased in the kidneys compared to the WTSHAM group (Figures 1B, C). This result indicated that renal RNLS expression was markedly reduced in CKD, which is consistent with previous studies (17, 30).

The thickness of the left ventricular posterior wall (LVPW) and interventricular septum (IVS) during systole and diastole was recorded as indexes of LVH. From 6 to 18 weeks, all these parameters showed upward trends in CKD mice, suggesting the progression of LVH (Figures 1D–G). At 18 weeks, LVPWs (*p* < 0.001) and IVS (*p* < 0.05) were significantly thicker in WTCKD mice compared to the WTSHAM group (Figures 1D–G), but fractional shortening (FS) and ejection fraction (EF) were not different (Figures 1H, I). Much more apparent trends of LVPW and IVS thickening were detected in KOCKD mice (Figures 1D–G). Significant differences in LVPW thickness were observed between KOCKD and WTCKD mice from the sixth week, and at 18 weeks, both LVPWs and IVS of KOCKD mice were significantly thicker than WTCKD mice (*p* < 0.001) (Figures 1D–G). FS and EF values of KOCKD mice increased continuously with the LVH progression and were higher than the other two groups at 18 weeks, although the differences were not significant (Figures 1H, I). These results showed that RNLS deficiency aggravated the pathologic LVH in CKD.

To examine the effect of RNLS knockout in normal non-pathological hearts, we additionally compared echocardiography data between the KOSHAM group and the WTSHAM group. The results were shown in Supplementary Figure 3. Briefly, the LVH index (LVPW and IVS thickness) in KOSHAM was significantly greater than the WTSHAM group at 18 weeks, while the renal functions were not different. These results indicated that RNLS might play an important role in maintaining normal heart structure.

### RNLS cardiac-specific overexpression alleviated cardiac remodeling induced by CKD

**FIGURE 2:**
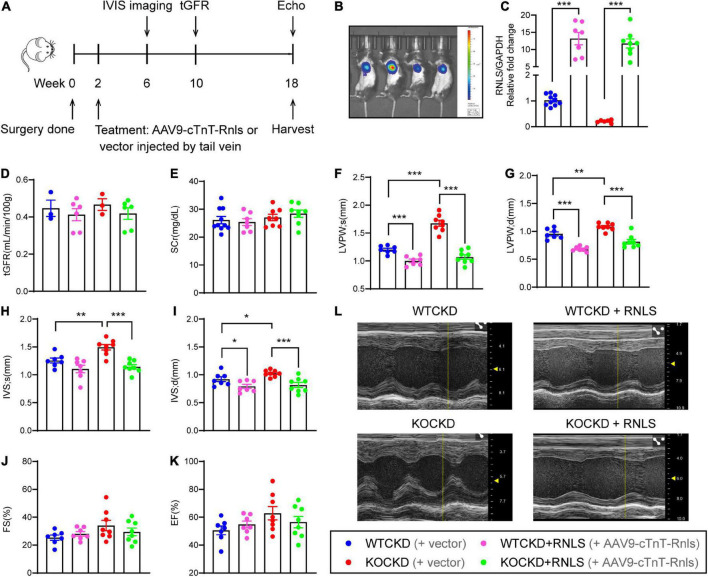
AAV9-cTnT-Rnls alleviated LVH hypertrophy in WTCKD and KOCKD mice. **(A)** Design of the experiment. WTCKD and KOCKD mice were injected with either AAV9-cTnT-Rnls or vector via tail vein at 2 weeks. The biodistribution of the virus was monitored by IVIS spectrum 4 weeks after injection (at 6 weeks). tGFR (ml/min/100 g) was detected at 10 weeks. Echocardiography was performed at 18 weeks, then mice were sacrificed. **(B)** Representative image using the *in vivo* Imaging System (IVIS) spectrum. A random representative was chosen from each group. **(C)** qPCR analysis of the mRNA expression of RNLS in the hearts. *n* = 6–10, ***p < 0.001. **(D)** tGFR measured by transdermal patch at 10 weeks. *n* = 3–6 per group. **(E)** Serum creatinine of CKD groups at 18 weeks. *n* = 6–8 per group. **(F–K)** Echocardiographic parameters, including LVPWs, LVPWd, IVSs, IVSd, FS, and EF at 18 weeks. WTCKD + vector group, *n* = 10; WTCKD + AAV9-cTnT-Rnls group, *n* = 7; KOCKD + vector group, *n* = 9; KOCKD + AAV9-cTnT-Rnls group, *n* = 8. Data were shown as mean ± SEM. *p < 0.05, **p < 0.01, ***p < 0.001. **(L)** Representative B-mode and M-mode echocardiographic images of each group. tGFR, transcutaneous glomerular filtration rate; LVPW, left ventricle posterior wall; IVS, interventricular septum; d, diastole; s, systole; FS, fractional shortening; EF, ejection fraction.

To determine the cardioprotective effects of RNLS independent of renal function, CKD mice were treated with AAV9-carrying RNLS under the control of the cardiomyocyte-specific cardiac troponin T (cTnT) promoter (AAV9-cTnT-Rnls) or the vectors at 2 weeks (Figure 2A). And 4 weeks later (at 6 weeks), we confirmed the dominant overexpression of RNLS in the heart by the *In Vivo* Imaging System (IVIS) spectrum (Figure 2B). Real-time PCR results showed that RNLS overexpression was persistent to 18 weeks, and it increased 5∼20 times RNLS expression in the hearts compared with controls (Figure 2C). No differences in renal function were observed among CKD groups at 2-time points (8 weeks and 16 weeks after the injections), indicating no effect of treatment on renal function (Figures 2D, E). While echocardiography data showed that RNLS overexpression made significant changes in LVPW thickness (Figures 2F, G). Cardiac-specific RNLS overexpression effectively inhibited the thickening of LVPW in WTCKD mice (*p* < 0.001) and significantly reversed the LVH phenotype exacerbated by RNLS knockout (all parameters of LVPW and IVS, *p* < 0.001) (Figures 2F–L). FS and EF showed no differences (Figures 2J, K). There were no statistically significant differences in LVPW and IVS thickness between WTCKD + RNLS and KOCKD + RNLS groups, and this may be because RNLS expression did not significantly differ between these two groups (Figure 2C). The entire echocardiography data were shown in Supplementary Figure 4.

**FIGURE 3:**
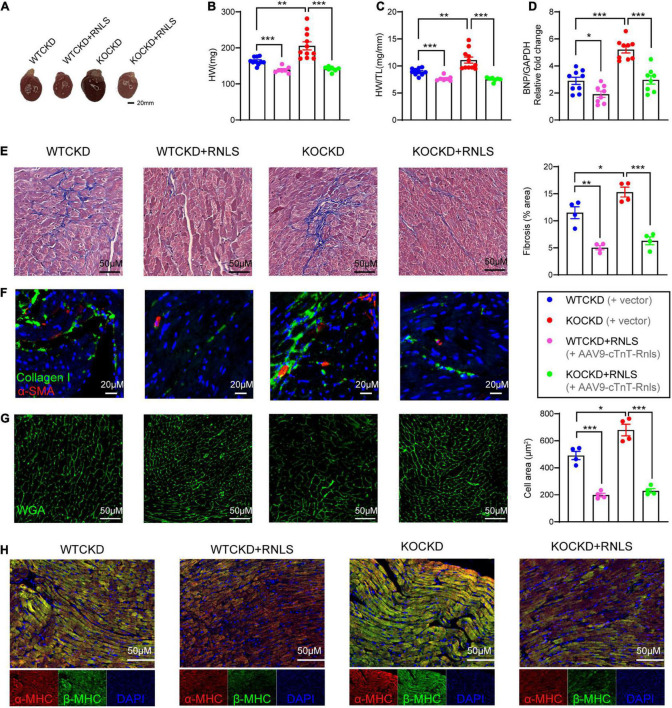
AAV9-cTnT-Rnls attenuated the pathologic features of myocardial fibrosis and myocardial hypertrophy in WTCKD and KOCKD mice. **(A)** Representative gross pictures of the heart. **(B,C)** All groups’ heart weight (HW) and heart weight to tibia length ratio (HW/TL). *n* = 7–10 per group, data were shown as mean ± SEM, **p < 0.01, ***p < 0.001. **(D)** qPCR analysis of the relative mRNA expression of BNP in the hearts. *n* = 8–9 per group, data were shown as mean ± SEM, *p < 0.05, ***p < 0.001. **(E)** Masson trichrome staining of the left ventricular posterior wall, quantification by Image J. (100×). **(F)** Immunofluorescence staining for collagen type 1 (green) and α-SMA (red). (200×). **(G)** Immunofluorescence staining for WGA (green), quantification by Image J. (100×). **(H)** Immunofluorescent staining for α-MHC (red), β-MHC (green), and DAPI (blue), as indicated. (100×) HW, heart weight; HW/TL, heart weight to tibia length ratio; BNP, B-type natriuretic peptide; α-SMA, α-smooth muscle actin; WGA, wheat germ agglutinin; MHC, myosin heavy chain.

At 18 weeks, hearts were harvested. The overall heart sizes were significantly reduced in groups with RNLS overexpression (Figure 3A), and so were the heart weights (Figure 3B) and the ratios of heart weight to tibia length (Figure 3C). Moreover, expression of brain natriuretic peptide (BNP) was more obviously elevated in KOCKD mice than WTCKD mice and was also significantly decreased by RNLS overexpression (Figure 3D).

To detect the pathological hallmarks of cardiac remodeling (myocardial interstitial fibrosis and cardiomyocyte hypertrophy), myocardial tissues were subjected to histopathological analysis. CKD caused massive interstitial fibrosis, and the situation was more severe in KOCKD mice (Figure 3E). But in groups with RNLS overexpression, we only observed mild fibrosis by Masson’s trichrome staining, indicating fibrosis was markedly reduced (WTCKD + RNLS vs. WTCKD *p* < 0.01, KOCKD + RNLS vs. KOCKD *p* < 0.001) (Figure 3E). Immunofluorescence showed that collagen I and α-SMA levels were also decreased by RNLS overexpression (Figure 3F). These results indicated that RNLS level impacted the degree of myocardial interstitial fibrosis. Wheat germ agglutinin (WGA) staining demonstrated that CKD induced remarkable cardiomyocyte hypertrophy, which was aggravated by RNLS deficiency (Figure 3G). In contrast, cardiac overexpression of RNLS significantly mitigated the cardiomyocyte hypertrophy and disordered arrangement caused by CKD (Figure 3G). β-MHC and the ratio of β-MHC to α-MHC are molecular markers of myocardial hypertrophy, which are often elevated in pathological circumstances (37). Similarly, β-MHC expression was highest in KOCKD mice, and RNLS overexpression reduced it obviously (Figure 3H). These results suggest that RNLS level affected the degree of cardiac fibrosis and cardiomyocyte hypertrophy, and overexpression of RNLS significantly alleviated cardiac remodeling induced by CKD.

### RNLS overexpression downregulated several ECM-related collagens and ECM organization and ECM-receptor interaction pathway

**FIGURE 4:**
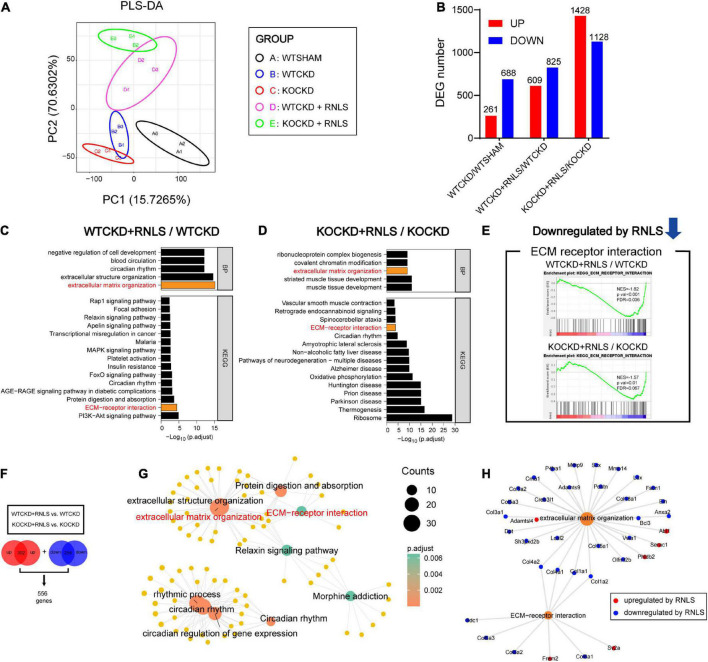
Overexpression of RNLS downregulated ECM organization and ECM-receptor interaction in CKD hearts. **(A)** Partial Least Squares Discriminatory Analysis (PLS-DA) score plot shows that groups are separated to, respectively, different clusters. *n* = 3 per group. **(B)** The number of differentially expressed genes (DEGs) in WTCKD/WTSHAM, WTCKD + RNLS/WTCKD, and KOCKD + RNLS/KOCKD (DEGs were selected by *p* < 0.05, |log2FC| > 0.32). **(C,D)** GOBP and KEGG analysis of DEGs in WTCKD + RNLS/WTCKD and KOCKD + RNLS/KOCKD. The top 5 of GOBP and the top 15 of KEGG pathways are shown. **(E)** GSEA revealed that the ECM-receptor interaction pathway was downregulated by RNLS overexpression. **(F)** The overlapped upregulated DEGs and downregulated DEGs between WTCKD + RNLS/WTCKD and KOCKD + RNLS/KOCKD clusters. **(G)** Diagram showing the top 5 items of the GOBP and KEGG analysis enriched by 556 DEGs in **(F)**. **(H)** Diagram showing DEGs enriched in ECM organization and ECM-receptor interaction. WTCKD, WTCKD + vector group; WTCKD + RNLS, WTCKD + AAV9-cTnT-Rnls group; KOCKD, KOCKD + vector group; KOCKD + RNLS, KOCKD + AAV9-cTnT-Rnls group. ECM, extracellular matrix.

To understand the underlying mechanisms, we carried out RNA-seq analysis. The Partial Least Squares Discriminatory Analysis (PLS-DA) score plot showed that groups were separated into different clusters, respectively (Figure 4A). Differentially expressed genes were selected by FC > 1.25 and *p* < 0.05, and the numbers of differentially expressed genes (DEGs) were shown in Figure 4B. Gene Ontology biological process (GOBP) and Kyoto Encyclopedia of Genes and Genomes (KEGG) enrichment analysis were performed on selected DEGs that were regulated by RNLS overexpression (WTCKD + RNLS/WTCKD, KOCKD + RNLS/KOCKD). Interestingly, two pathways were both significantly enriched, which were extracellular matrix (ECM) organization from GOBP (top 5) and ECM-receptor interaction from KEGG (top 15) (Figures 4C, D). Moreover, gene set enrichment analysis (GSEA) also revealed that the ECM-receptor interaction pathway was significantly downregulated by RNLS overexpression both in WTCKD and KOCKD mice (Figure 4E).

Consistent 556 DEGs were obtained by taking the intersection of the two clusters (WTCKD + RNLS/WTCKD, KOCKD + RNLS/KOCKD), including 302 upregulated and 254 downregulated (Figure 4F). The 5 top GOBP and KEGG pathways that were enriched by these 556 DEGs were shown in Figure 4G. ECM organization and ECM-receptor interaction were still significantly enriched (Figure 4G). In these two pathways, several members of the collagen family, which are closely linked to cardiac fibrosis, were downregulated by RNLS overexpression, such as COL1A1, COL1A2, COL3A1, COL4A1 and COL4A2 (Figure 4H). These results revealed that RNLS overexpression reduced the expressions of several collagens and downregulated related ECM-receptor interaction pathway and ECM organization.

### CKD-induced fibroblast proliferation and EMT were inhibited by RNLS overexpression

**FIGURE 5:**
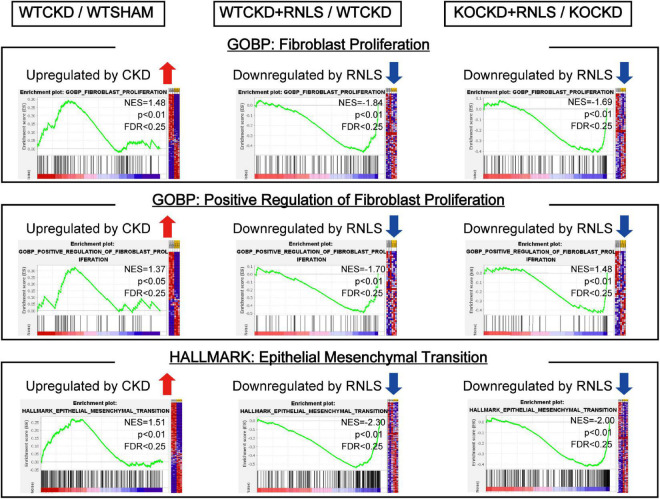
Gene set enrichment analysis (GSEA) using the transcriptome data. GSEA revealed fibroblast proliferation, positive regulation of fibroblast proliferation, and epithelial-mesenchymal transition (EMT) were upregulated by CKD. In contrast, they were downregulated by RNLS overexpression in WTCKD + RNLS/WTCKD and KOCKD + RNLS/KOCKD. Detailed results of GSEA are shown in Table 1.

To understand what functional differences RNLS made in phenotypic reversion, we performed GSEA to test the tendency of all DEGs from the whole transcriptomes. Data from every two groups (WTCKD vs. WTSHAM, WTCKD + RNLS vs. WTCKD, and KOCKD + RNLS vs. KOCKD) were submitted to GSEA software utilizing GOBP and hallmark gene sets. Overlapped gene sets (significantly enriched by |NES| > 1; *p* < 0.05; FDR < 0.25) are shown in Table 1. It is interesting to note that they were mostly related to cardiac fibrosis including fibroblast proliferation, migration of immune cells, antigen processing and presentation, cytokine production, and epithelial-mesenchymal transition (EMT) (Table 1). Epicardial cells undergo EMT to produce cardiac fibroblasts (38). Cardiac fibroblasts are the main effector cells in the development of cardiac fibrosis by over-proliferation and increasing fibrous ECM proteins (39). As expected, fibroblast proliferation, positive regulation of fibroblast proliferation, and EMT were upregulated by CKD. In contrast, these gene sets were downregulated by RNLS overexpression both in WTCKD and KOCKD mice, showing that RNLS reversed cardiac fibrosis induced by CKD mainly through affecting cardiac fibroblasts (Figure 5).

**TABLE 1:** GSEA results using the MSigDB GOBP and hallmark databases.

	Upregulated by CKD			Downregulated by RNLS^1^			Downregulated by RNLS^2^		
Gene sets	NES	NOM *p*-val	FDR	NES	NOM *p*-val	FDR	NES	NOM *p*-val	FDR
GOBP_FIBROBLAST_PROLIFERATION	1.481	0.000	0.157	−1.845	0.000	0.032	−1.694	0.000	0.095
GOBP_REGULATION_OF_FIBROBLAST_PROLIFERATION	1.444	0.000	0.174	−1.603	0.000	0.088	−1.446	0.000	0.245
GOBP_POSITIVE_REGULATION_OF_LEUKOCYTE_MIGRATION	1.431	0.000	0.183	−1.871	0.000	0.028	−1.465	0.000	0.239
GOBP_POSITIVE_REGULATION_OF_FIBROBLAST_PROLIFERATION	1.373	0.036	0.222	−1.704	0.000	0.060	−1.672	0.000	0.108
GOBP_ANTIGEN_PROCESSING_ AND_PRESENTATION_OF_PEPTIDE_ANTIGEN	1.581	0.000	0.109	−1.483	0.029	0.136	−1.847	0.000	0.036
GOBP_ANTIGEN_PROCESSING_AND_PRESENTATION_OF_EXOGENOUS_PEPTIDE_ANTIGEN	1.436	0.040	0.180	−1.531	0.049	0.116	−1.839	0.000	0.037
GOBP_MACROPHAGE_CYTOKINE_PRODUCTION	1.483	0.020	0.157	−1.552	0.035	0.108	−1.538	0.028	0.183
GOBP_MACROPHAGE_MIGRATION	1.493	0.018	0.151	−1.578	0.000	0.096	−1.512	0.024	0.206
GOBP_ANTIGEN_PROCESSING_AND_PRESENTATION_OF_EXOGENOUS_ANTIGEN	1.660	0.018	0.081	−1.471	0.000	0.141	−1.655	0.024	0.112
HALLMARK_EPITHELIAL_ MESENCHYMAL_TRANSITION	1.509	0.002	0.022	−2.300	0.000	0.000	−2.005	0.000	0.000
HALLMARK_MYOGENESIS	2.197	0.000	0.000	−1.439	0.008	0.040	−1.232	0.045	0.119

Upregulated by CKD: WTCKD + vector group vs. WTSHAM group. Downregulated by RNLS^1^: WTCKD + AAV9-cTnT-Rnls group vs. WTCKD + vector group. Downregulated by RNLS^2^: KOCKD + AAV9-cTnT-Rnls group vs. KOCKD + vector group. |NES| > 1, *p* < 0.05, FDR < 0.25 were raised in table. NES, normalized enrichment score; NOM *p*-val, nominal *p-value*; FDR, false discovery rate.

### RNLS reduced proliferation and the expression of α-SMA in cardiac fibroblasts stimulated by TGF-β

**FIGURE 6:**
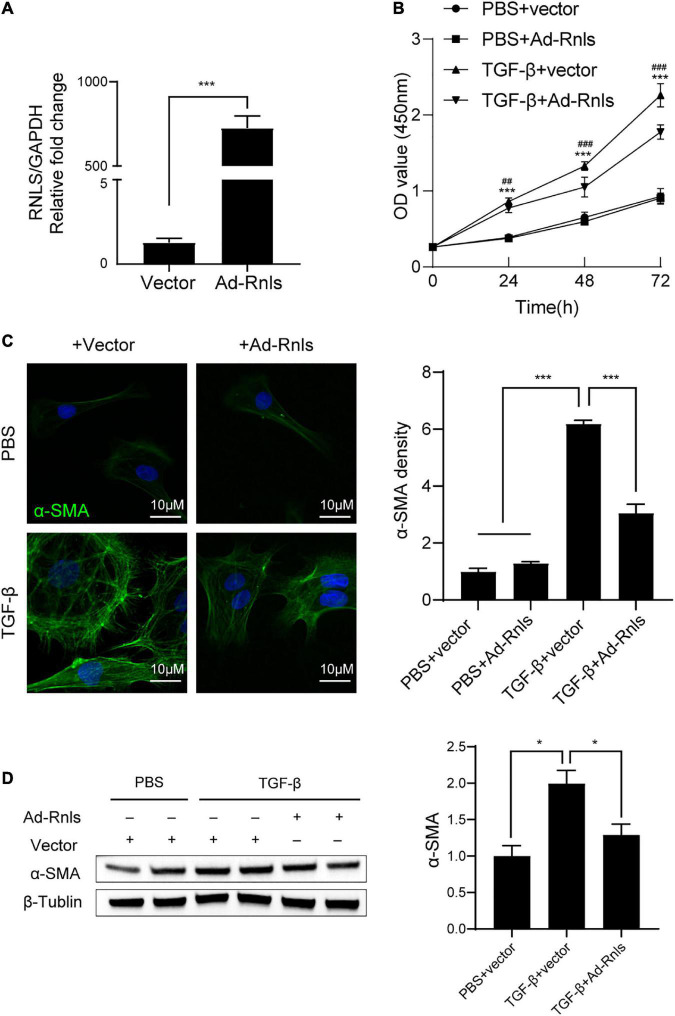
RNLS overexpression inhibited cardiac fibroblasts (CFs) proliferation and the expression of α-SMA stimulated by TGF-β. **(A)** qPCR analysis of the relative mRNA expression of RNLS. **(B)** CF proliferation detected by cell counting kit-8 (CCK8) assay. CFs were transfected with Ad-RNLS or vector and followed by stimulated with TGF-β for 24, 48, 72 hrs. ***p < 0.001, ^##^ *p* < 0.01, ^###^*p* < 0.001. * vs. PBS + vector group, ^#^ vs. TGF-β + Ad-Rnls group. **(C)** Immunofluorescence staining for α-SMA (green) and DAPI (blue) in CFs, quantification by Image J. (400×) ***p < 0.001. **(D)** Representative western blot analysis and its quantification. *p < 0.05. **(C,D)** CFs were transfected with Ad-RNLS or vector and followed by stimulated with TGF-β for 24 h.

To validate the above results, we conducted *in vitro* experiments using primary cardiac fibroblasts (CFs) isolated from neonatal rat hearts. CFs were transfected with adenovirus containing RNLS gene (ad-Rnls). RT-qPCR results showed that ad-Rnls enhanced the mRNA expression of RNLS by more than 500-fold over the vector group (Figure 6A). After transfection, the cells were stimulated with TGF-β for 24h, 48h, and 72h. The cellular proliferation was detected by cell counting kit-8 (CCK8) assay. As shown in Figure 6B, TGF-β promoted CF proliferation after 24 h of stimulation (*p* < 0.001), but overexpression of RNLS significantly inhibited the promotion effect of TGF-β (*p* < 0.01) (Figure 6B). Similar results were observed at 48h and 72h. More than that, immunofluorescence and western blot results showed that RNLS significantly reduced the expression of α-SMA in CFs stimulated with TGF-β for 24h (Figures 6C, D). These results indicated that RNLS overexpression inhibited the proliferation and the expression of α-SMA in CFs with TGF-β stimulation.

## Discussion

In the present study, we confirmed that RNLS deficiency significantly accelerated the progression of cardiac remodeling in CKD, whereas RNLS cardiac-specific overexpression reversed this cardiac phenotype both in WTCKD and KOCKD mice. The following RNA-seq analysis revealed that RNLS exerted protective effects primarily by reducing cardiac fibrosis, and *in vitro* experiments confirmed that RNLS inhibited the proliferation and the expression of α-SMA in cardiac fibroblasts stimulated by TGF-β. Therefore, our study suggests that RNLS is a potential therapeutic factor for pathological cardiac remodeling.

In this study, no significant differences in EF values were observed at the end of the experiments (18 weeks after surgery) between control and CKD mice, suggesting that heart function may still be in a compensatory state. Instead, two main characteristics of cardiac remodeling were observed in CKD mice, which were left ventricular hypertrophy and cardiac fibrosis, and both of them were reduced by RNLS cardiac-specific overexpression (Figures 1–3). It is well-known that cardiac hypertrophy and fibrosis are mutually promoting in the progression of cardiac remodeling (40, 41). Cardiomyocytes can promote cardiac fibrosis by activating pro-fibrogenic programming (39, 42), and CFs promote cardiomyocyte hypertrophy by secreting growth cytokines in pathological conditions (43, 44). In this study, analysis performed for RNA-seq data revealed that RNLS was mainly involved in the mechanisms and pathways of cardiac fibrosis, such as ECM organization, ECM receptor interaction, fibroblast proliferation, and EMT (Figures 4, 5). CFs are a major cell type of cardiac tissues (45), which can be derived from the epicardium through EMT (46, 47), and myofibroblasts, the activated CFs, are the main effector cells to secrete ECM proteins in a remodeling heart (39, 48, 49). Therefore, we next validated the direct effects of RNLS on primary CFs *in vitro*, and the results confirmed that RNLS significantly inhibited CFs proliferation and downregulated the expression of α-SMA in TGF-β stimulated CFs (Figure 6). We thus believe that RNLS has great therapeutic potential for the heart by antagonizing fibrotic mechanisms induced by CKD.

Since it was identified, RNLS has been proven to be a beneficial factor for many cardiovascular or renal diseases because of its enzymatic or non-enzymatic functions (50). Some studies, which suggest RNLS protects the cardiovascular system through its enzymatic functions, believe RNLS can modulate blood pressure (BP) by metabolizing catecholamines (11, 18, 19, 51). Although this view has been challenged, we found that RNLS knockout influenced BP in mice, which is consistent with previous evidence (23, 52). In this study, we observed that knockout of RNLS led to higher systolic blood pressure (SBP) (KOSHAM vs. WTSHAM, 129.30 ± 6.51mmHg vs. 119.29 ± 9.5 mmHg, *p* < 0.05) (Supplementary Figure 5A). However, in CKD, there was no significant group difference in SBP between KOCKD and WTCKD groups from the sixth-week post-surgery (average BP > 135mmHg) (Supplementary Figure 5B). Thus, the alteration of BP caused by RNLS knockout maybe not the principal contributing factor for the more severe cardiac remodeling phenotype in KO mice. In addition, cardiac-specific RNLS overexpression had little effect on hypertension, but significantly reduced the thickness of LVPW and IVS in CKD mice (Supplementary Figure 5B). Therefore, we suggest that RNLS has direct protective effects on hearts independent of modulating BP.

Indeed, recent studies of RNLS have already focused on its non-enzymatic functions (53–55). Evidence has shown that RNLS functions as a signaling factor exerting potent pro-survival and anti-inflammatory effects to protect cells, tissues, and organs (12, 22, 56–58). In recent years, numerous clinical studies have reported that RNLS seems to be a promising biomarker and a potential therapeutic target in cardiovascular diseases (59–62). In this study, we provided clear data demonstrating that RNLS alleviated CKD-induced pathological cardiac fibrosis, and the possible mechanisms involved are as below: (i) RNLS significantly inhibited CKD-induced EMT, one source that CFs derived from Figure 5 and Table 1. (ii) In the development of cardiac fibrosis, CF over-proliferation is induced and CFs transdifferentiate into myofibroblasts, which express α-SMA and secrete ECM proteins (39). Our results showed that RNLS significantly inhibited CFs proliferation and the expression of α-SMA *in vivo* and *in vitro* (Figures 3, 5, 6). (iii) Moreover, genes relating to ECM proteins, such as COL1A1, COL3A1, COL4A1, COL4A2, POSTN, FSCN1, and BCL3, were significantly downregulated by RNLS (Figure 4). (iv) In addition to the effects on CFs, RNA-seq analysis also revealed that RNLS downregulated leukocyte and macrophage migration, macrophage cytokine production, and antigen presentation (Table 1). All the above mechanisms are proven to be promoted by the TGF-β pathway in cardiac fibrosis (39, 42). And our RNA-seq results indicated that RNLS-regulating DEGs were significantly enriched in several pathways that involved in non-Smad TGF-β pathways, such as PI3K/Akt and the mitogen-activated protein kinases (MAPKs) pathway (Figure 4C), which is consistent with previous studies (14, 22, 63). We hypothesized that the potential molecular mechanisms may be related to these pathways, but more experimental studies are needed.

There were a few limitations in the present study. We found RNLS overexpression significantly reduced cardiomyocyte hypertrophy *in vivo* and RNA-seq analysis showed that RNLS downregulated the hallmark myogenesis gene set, but we didn’t validate it *in vitro*. Secondly, we overexpressed RNLS using the cardiomyocyte-specific cTnT promoter, but whether RNLS can be secreted from cardiomyocytes into CFs and play an anti-fibrotic role should be confirmed. These limitations will be investigated in follow-up studies, together with the underlying molecular mechanisms involved.

In summary, by using knockout mice, we confirmed the important role of RNLS in CKD-induced cardiac remodeling, and we found that RNLS overexpression alleviated LVH and fibrosis in CKD mice mainly through an anti-fibrotic mechanism. Our finding implies that RNLS may be a valuable therapeutic target of cardiac treatment for CKD patients, and its functional roles deserve further investigation.

## Data availability statement

The datasets presented in this study can be found in online repositories. The names of the repository/repositories and accession number(s) can be found below: https://www.ncbi.nlm.nih.gov/geo/, GSE199350.

## Ethics statement

The animal study was reviewed and approved by the Institutional Animal Care and Use Committee of Shanghai Jiao Tong University Affiliated Sixth People’s Hospital.

## Author contributions

NW and JY conceived and supervised the study, acquired funding, and revised the manuscript. YW, LB, FZ, SG, and JW performed the animal study and/or contributed materials. YW performed the data analysis and wrote the manuscript draft. All authors contributed to the article and approved the submitted version.
