# Supplementary material for: Cardiac-specific renalase overexpression alleviates CKD-induced pathological cardiac remodeling in mice
Source: Front Cardiovasc Med. 2022 Dec 15;9:1061146. doi: 10.3389/fcvm.2022.1061146 (PMC9798007; doi:10.3389/fcvm.2022.1061146)
Supplement: Supplementary file 1 [file Data_Sheet_1.pdf]

Supplementary Material

1 Supplementary method

1.1 Blood pressure

Blood pressure was measured by a computerized tail-cuff system (Kent Scientific, Torrington, CT, USA) in conscious animals. Animals were trained for 3 consecutive days to become accustomed to the procedure. Each time animals were put in the restrainer for 15 minutes before measurements. For each animal, at least 10 measurements were recorded to calculate mean blood pressure and heart rate, and the results were averaged for each animal for 5 consecutive days. The mean values were used for comparisons.

1.2 RNA-seq analysis

Raw data (raw reads) were processed using Trimmomatic. The reads containing ploy-N and the low-quality reads were removed to obtain the clean reads. Then the clean reads were mapped to mm9 using hisat2. FPKM value of each gene was calculated using cufflinks, and the read counts of each gene were obtained by htseq-count. DEGs were identified using the DESeq (2012) R package functions estimate Size Factors and nbinom Test. P value < 0.05 and foldchange >1.25 set as the threshold for significantly differential expression. Hierarchical cluster analysis of DEGs was performed to explore gene expression patterns. GO enrichment and KEGG pathway enrichment analysis of DEGs were respectively performed using R based on the hypergeometric distribution.

1.3 Quantitative real-time PCR

Data were expressed at an amplification number of 2-ΔΔCt by normalization of GAPDH and compar ison of controls. Primers used for qPCR are as follows: msRNLS forward: AAGCCTCTGACATCC CCCAT, reverse: GGGAGACTTCTGCACCTGACTT; msBNP forward: TTTGGGCTGTAACGCA CTGA, reverse: CACTTCAAAGGTGGTCCCAGA; msGAPDH forward: TGTTCCTACCCCCAAT GTGTC, reverse: TGAAGTCGCAGGAGACAACC; rRNLS forward: GGACAAGGCTGGGGACA TAG, reverse: TGGTGCTTTTTGGCATAATGAG; rGAPDH for-ward: GGTGCTGAGTATGTCG TGGAG reverse: TTGCTGACAATCTTGAGGGAG.

2 Supplementary Figures

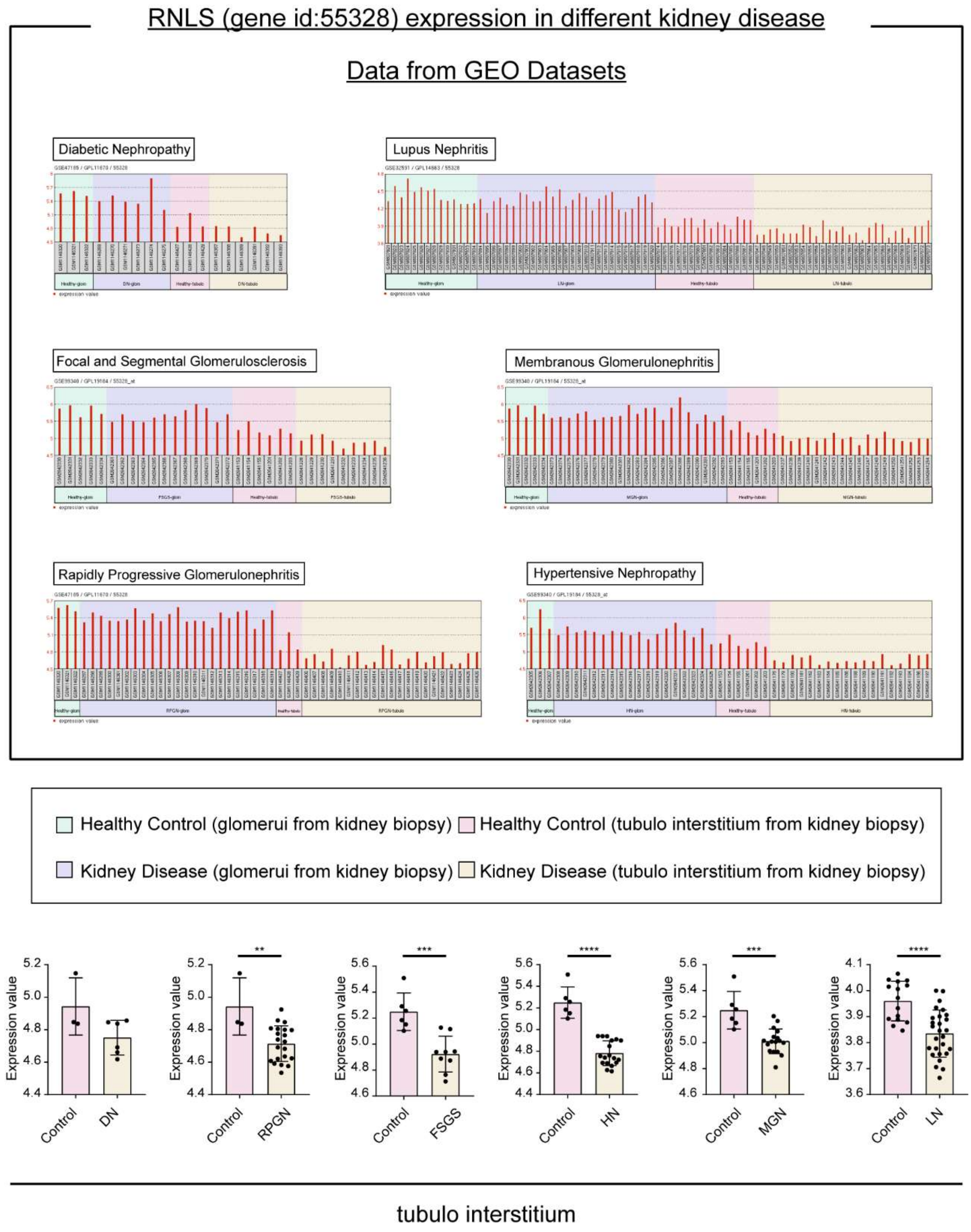

Supplementary Figure 1 RNLS expression in human kidney biopsy from patients with different kidney diseases. Data from the DEO database. Data were shown as mean ± SEM. *p<0.05 **p<0.01 ***p<0.001 **** p<0.0001.

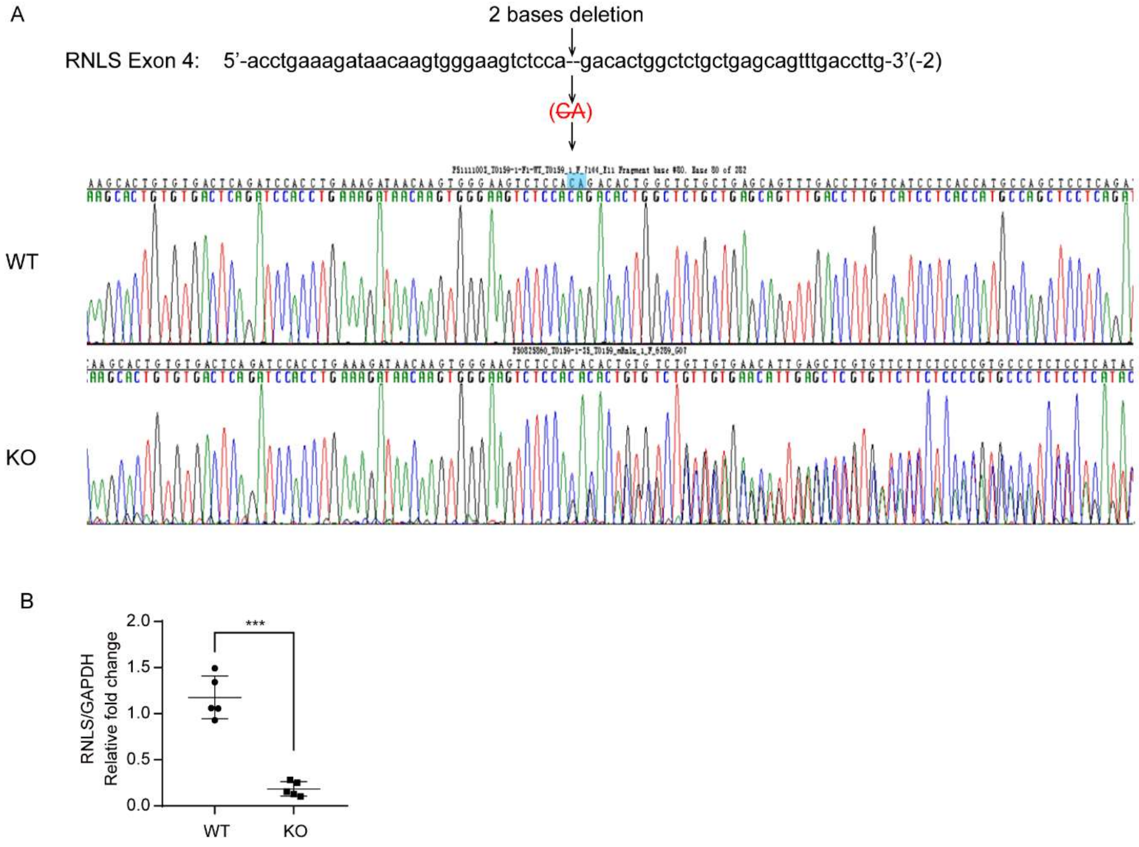

Supplementary Figure 2 RNLS KO mice were generated by Transcription activator-like effector nuclease (TALEN)-mediated gene targeting in C57BL/6J strain mice. A. Exon 4 of RNLS was

selected as the TALEN target site. B. Validation of RNLS expression by qPCR. Data were shown as mean ± SEM. *p<0.05 **p<0.01 ***p<0.001.

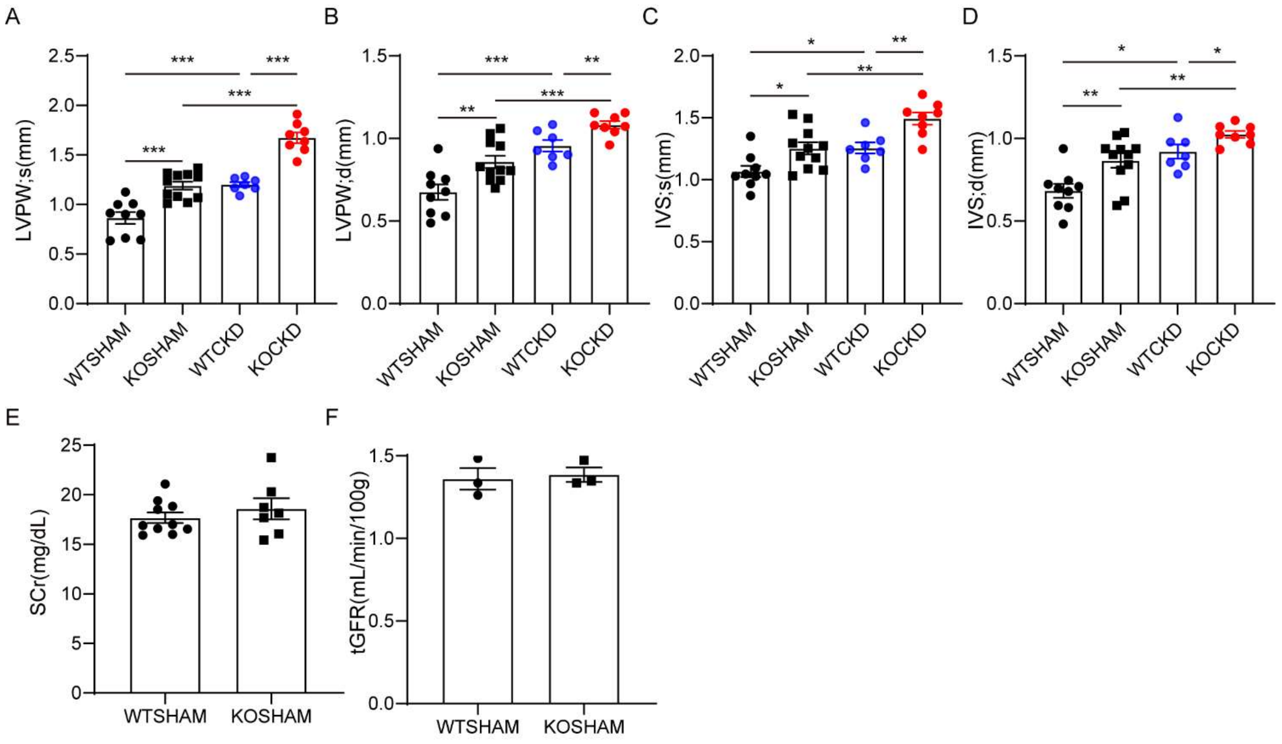

Supplementary Figure 3 A-E Echocardiographic parameters of WTSHAM group, KOSHAM group, WTCKD group, and KOCKD group, including LVPWs, LVPWd, IVSs, IVSd, at week 18. E tGFR was measured by transdermal patch at 10 weeks. F Serum creatinine of SHAM groups at 18 weeks. WTSHAM group, n = 9; KOSHAM group, n = 11; WTCKD+vector group, n = 10;

WTCKD+AAV9-cTnT-RNLS group, n = 7; KOCKD+vector group, n = 9; Data were shown as mean ± SEM. *p<0.05 **p<0.01 ***p<0.001.

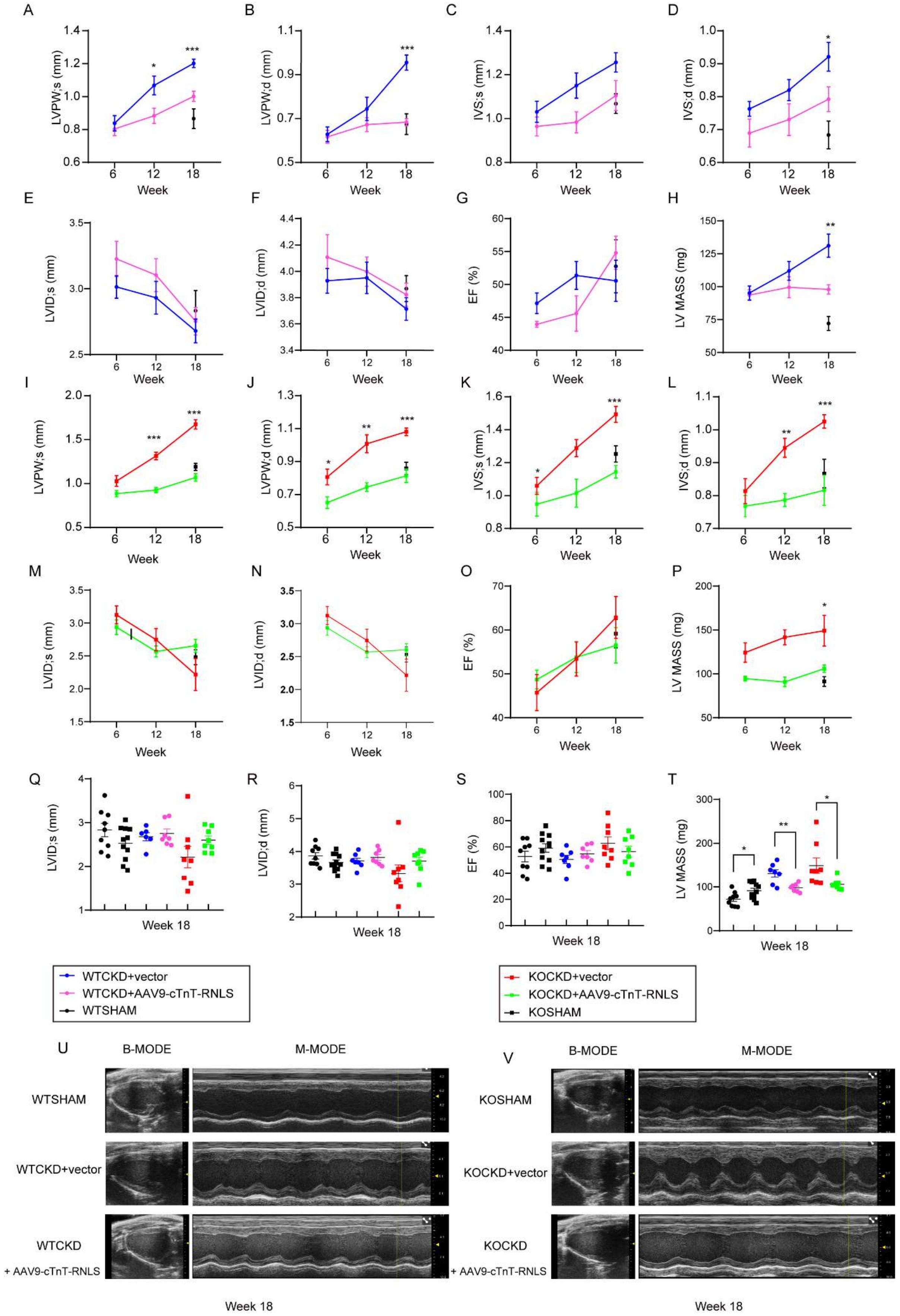

A B C D
LVPW;s (mm)
LVPW;d (mm)
IVS;s (mm)
IVS;d (mm)
Week
E F G H
LVID;s (mm)
LVID;d (mm)
EF (%)
LV MASS (mg)
I J K L
M N O P
Q R S T
Week 18
WTCKD+vector
WTCKD+AAV9-cTnT-RNLS
WTSHAM
KOCKD+vector
KOCKD+AAV9-cTnT-RNLS
KOSHAM
U
V
B-MODE
M-MODE
WTSHAM
WTCKD+vector
WTCKD
+ AAV9-cTnT-RNLS
KOSHAM
KOCKD+vector
KOCKD
+ AAV9-cTnT-RNLS
Week 18

Supplementary Figure 4 A-H Echocardiographic parameters, including LVPWs, LVPWd, IVSs, IVSd, LVIDs, LVIDd, EF, and LVMASS at week 6, week 12, and week 18. Q-T Echocardiographic parameters, including LVIDs, LVIDd, EF, and LVMASS at week 18. U-V Representative B-mode and M-mode echocardiographic images of each group. WTSHAM group, n = 9; KOSHAM group, n = 11; WTCKD+vector group, n = 10; WTCKD+AAV9-cTnT-RNLS group, n = 7; KOCKD+vector

group, n = 9; KOCKD+AAV9-cTnT-RNLS group, n = 8. Data were shown as mean ± SEM. *p<0.05 **p<0.01 ***p<0.001. *vs. WTCKD+vector or KOCKD+vector

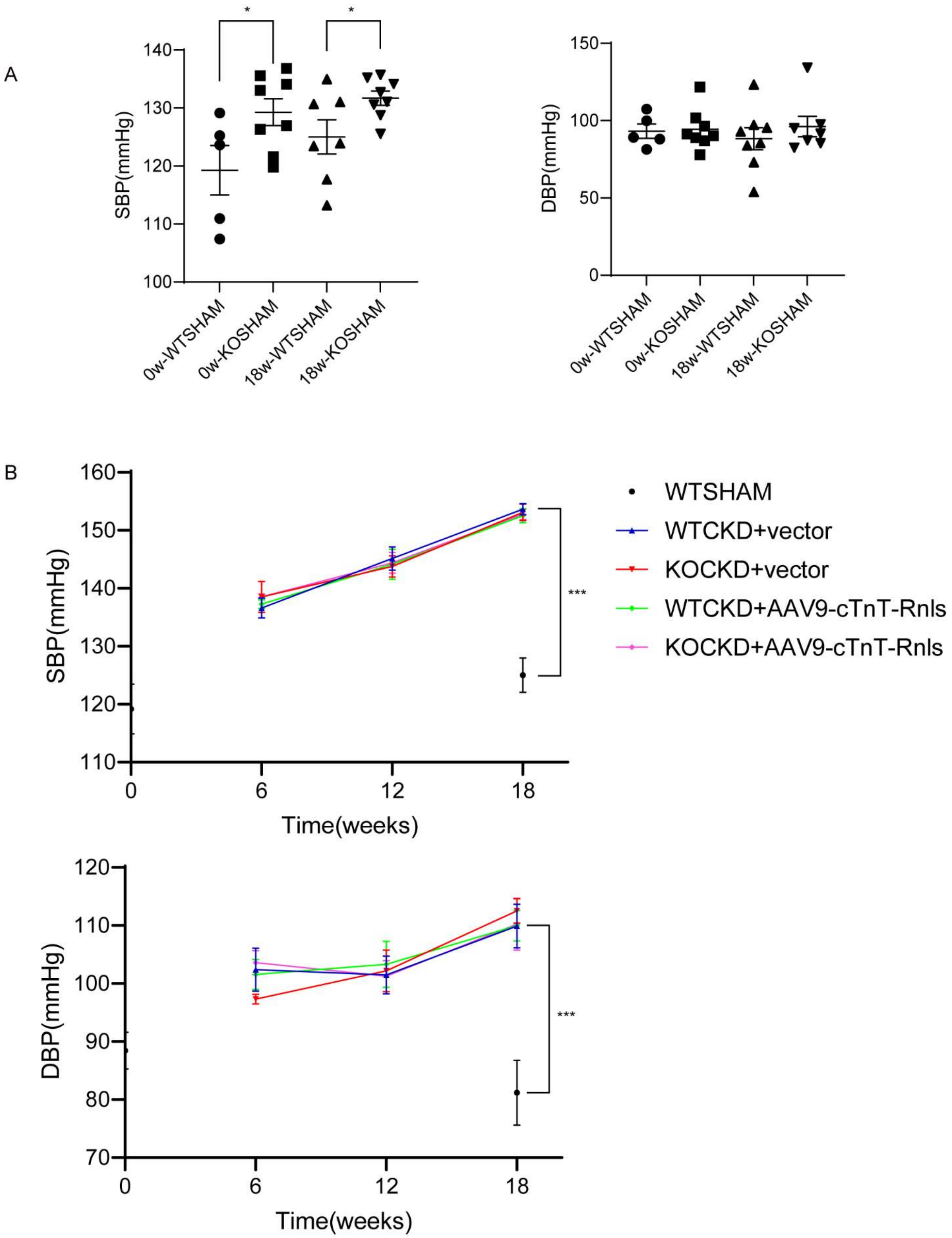

Supplementary Figure 5 A. Systolic and diastolic blood pressures of KOSHAM and WTSHAM groups. n = 5-8 per group. *p<0.05. B. Systolic and diastolic blood pressures of CKD and WTSHAM groups. n = 5-8 per group. Data were shown as mean ± SEM. ***p<0.001. *vs. WTSHAM group.
